# Effect of Pullulan Nanoparticle Surface Charges on HSA Complexation and Drug Release Behavior of HSA-Bound Nanoparticles

**DOI:** 10.1371/journal.pone.0049304

**Published:** 2012-11-14

**Authors:** Xiaojun Tao, Qiufang Zhang, Kai Ling, Yicun Chen, Wenzhi Yang, Fenfei Gao, Ganggang Shi

**Affiliations:** 1 Department of Cardiovascular Diseases, First Affiliated Hospital, Shantou University Medical College, Shantou, Guangdong, China; 2 Laboratory of Chinese Herbal Pharmacology, Renmin Hospital of Hubei University of Medicine, Shiyan, Hubei, China; 3 Department of Pharmacology, Shantou University Medical College, Shantou, Guangdong, China; 4 Institute of Biomedical Engineering, Chinese Academy of Medical Science & Peking Union Medical College, The Key Laboratory of Biomedical Material of Tianjin, Tianjin, People's Republic of China; University of Hyderabad, India

## Abstract

Nanoparticle (NP) compositions such as hydrophobicity and surface charge are vital to determine the presence and amount of human serum albumin (HSA) binding. The HSA binding influences drug release, biocompatibility, biodistribution, and intercellular trafficking of nanoparticles (NPs). Here, we prepared 2 kinds of nanomaterials to investigate HSA binding and evaluated drug release of HSA-bound NPs. Polysaccharides (pullulan) carboxyethylated to provide ionic derivatives were then conjugated to cholesterol groups to obtain cholesterol-modified carboxyethyl pullulan (CHCP). Cholesterol-modified pullulan (CHP) conjugate was synthesized with a similar degree of substitution of cholesterol moiety to CHCP. CHCP formed self-aggregated NPs in aqueous solution with a spherical structure and zeta potential of −19.9±0.23 mV, in contrast to −1.21±0.12 mV of CHP NPs. NPs could quench albumin fluorescence intensity with maximum emission intensity gradually decreasing up to a plateau at 9 to 12 h. Binding constants were 1.12×10^5^ M^−1^ and 0.70×10^5^ M^−1^ to CHP and CHCP, respectively, as determined by Stern-Volmer analysis. The complexation between HSA and NPs was a gradual process driven by hydrophobic force and inhibited by NP surface charge and shell-core structure. HSA conformation was altered by NPs with reduction of α-helical content, depending on interaction time and particle surface charges. These NPs could represent a sustained release carrier for mitoxantrone *in vitro*, and the bound HSA assisted in enhancing sustained drug release.

## Introduction

Nanomaterials hold great promise for use in drug delivery, as image contrast agents, and for diagnostic purposes [1]–[3]. Nanoscale objects in the form of capsules, liposomes, and particles are widely used for delivering small-molecular-weight drugs and macromolecular-protein drugs by localized or targeted delivery to the tissue of interest [4]–[7]. Polymeric amphiphiles such as polysaccharides modified with cholesterol groups can spontaneously form the self-aggregated NPs, which have shown huge potential for controlled drug release and targeting ability in medical and pharmaceutical application [8]–[10].

Despite the remarkable advances in nanoscience, surprisingly little is known about the responses of biological systems to NPs [11], [12]. NPs become coated with a layer of adsorbed proteins immediately upon contact with the physiological environment [13]. NPs entering the bloodstream initially bind abundant serum proteins, primarily HSA, making the NP**–**HSA complex the actual biological entity [14]–[17]. HSA binding influences the uptake and intercellular trafficking of NPs and affects particle biodistribution, biocompatibility, and therapeutic efficacy [18], [19]. HSA is the principal extracellular protein as it is responsible for transporting many exogenous and drugs *in vivo*. It has a high affinity for some antitumor drugs such as mitoxantrone, often chosen as model drug to assess the potential of NPs [20]–[22]. HSA binding inevitably influences the release of NP-based drugs because of the mutual adsorption among the drug, HSA and NPs. NPs, as a promising drug carrier, show controlled-release properties related to biodegradability, pH, ion, and temperature sensibility of materials [8]. To further explore drug-loaded NPs in the body for medical application, the effect of HSA binding on drug release must be investigated. Especially, we lack reports about the drug release behavior of HSA-bound NPs and the interaction among HSA, hydrophobic anticancer drugs, and polysaccharide-based NPs.

HSA binding is a key factor for determining the *in vivo* fate of intravenously administered colloidal drug carriers, which strongly depends on NP surface characteristics; indeed, plasma protein absorption increases with increasing surface charge density [23], [24]. Many attempts have been made to modify NP surface chemical composition to optimize polymer NP characteristics [25]–[27]. Nanomaterials can be designed to combine targeting proteins with a high efficiency in drug delivery [12], [28]–[29]. We investigated 2 types of NPs (maintaining hydrophobicity approximately constant) with different surface charges to examine the influence of particle surface charges on HSA binding.

Carboxyl groups are always conjugated to polymeric amphiphiles to form NPs with negative surface charge [30], [31]. In this work, we synthesized carboxyethyl pullulan (CEP) with additive reaction using acrylic acid and pullulan. Then, cholesterol was introduced into CEP to form cholesterol-modified carboxyethyl pullulan (CHCP) conjugates with esterification reaction. The prepared CHCP NPs were characterized by zeta potentiometry, dynamic light scattering and transmission electron microscopy to determine particle composition (surface charge, size, and shape). We also characterized cholesterol-modified pullulan (CHP) NPs, which consist of a hydrophobic core and hydrophilic shell: the hydrophobic core allows for encapsulation of hydrophobic substances, and the hydrophilic shell provides solubility and stability in aqueous solution [32]. CHP nanogels can form complex NPs with various proteins embedded in the NP core rather than adsorbed to the NP surface [33], [34]. NP complex formation with bovine serum albumin (BSA) involved 2 processes: a fast pre-equilibrium of loose binding and a slow tight inclusion of BSA into the hydrogel network [35]–[37]. Because of the structural homology of HSA and BSA, the CHP–HSA complexation may be as complicated as the CHP–BSA complexation.

We describe the effect of particle hydrophobicity and surface charge on the HSA binding to fully understand the NP–protein complexation. Structural change of HSA after NP binding was studied by fluorescent spectra and circular dichroism (CD) analysis. We loaded mitoxantrone into NPs to evaluate the drug release behavior *in vitro*. HSA was added to the release media to investigate drug release of the bound NPs. The HSA binding influenced the drug release characteristic of NPs, which is meaningful for these drug carriers to be further investigated for *in vivo* efficacy.

## Materials and Methods

### Materials

CHP was synthesized as described [38]. Pullulan (molecular weight: 20 kDa) was substituted with 3.11 cholesterol moieties per 100 glucose units. HSA (fatty acid free) was from Sigma-Aldrich Co (St Louis. MO, USA). Mitoxantrone was from Beijing Xinze Science and Technology Co. N-hydroxyl succinimide (NHS) was from Sigma without further purification. All other chemical reagents were of analytical grade and obtained from commercial sources.

### Synthesis of CHCP

Synthesis of CEP: Pullulan (3.0 g) was mixed with acrylic acid (1.26 mL) at molar ratio 1∶1. Then, the mixture was incubated for 4 h at 50°C with KOH solution as a catalytic agent. The reaction solution was cooled to room temperature and was placed into 500 mL ethanol. Yellowish-brown precipitation was obtained on removing the ethanol solution. It was dissolved with 40 mL distilled water, filtered, and dialyzed against 5000 mL hydrochloric acid solution (pH =  4.5±0.2) for 2 days and distilled water for 1 day. The CEP dialysate was then freezed-dried, resulting in a white cotton solid.

Synthesis of CHCP: cholesterol succinate (CHS) and NHS-activated cholesterol succinate (CSN) were synthesized according to the previously reported method [39], [40]. CEP was dissolved in 10 mL DMSO, and placed in oil bath pan with stirring at 45°C. CSN (CSN/glucose unit =  0.1∼0.5 mmol/mmol) and (1-(3-Dimethylaminopropyl)-3-ethylcarbodiimide hydrochloride (EDC/CSN = 1.0 mmol/mmol) were dissolved in the mixture of DMSO and tetrahydrofuran. The mixture was dropped into the prepared CEP solution, and activated at 45°C for 72 h. The reactant mixture was then put into ethanol. The precipitate was collected by filtration and sequentially washed with ethanol, tetrahydrofuran, and diethyl ether. The sample was dried under vacuum to obtain CHCP conjugate.

### Fourier transform infrared (FT-IR) spectroscopy and nuclear magnetic resonance (NMR)

The FT-IR spectra of pullulan, CEP, and CSN were obtained as KBr pellets for FT-IR spectroscopy (Nicolet NEXUS 470-ESP, USA) at room temperature. The chemical structure of CHCP was confirmed by 500 MHz H^1^-NMR (CDCl3 with TMS and DMSO-*d6*), which was also used to calculate the degree of substitution (DS) of cholesterol and carboxyethyl residues per 100 glucose units in pullulan.

### Preparation and characterization of NPs

CHCP and CHP conjugates were both dispersed in water under gentle shaking at 37°C for 48 h, and underwent sonication with a probe type sonifier (Automatic Ultrasonic Processor UH-500 A, China) at 100 W for 2 min. The mean sizes of obtained particles were determined by dynamic light scattering with a BI-90US (Japan) light-scattering spectrophotometer. The NP zeta potential was measured using a zeta potentiometer (Zetasizer 3000 HS, Malvern Instruments Ltd, Malvern UK) operated at 11.4 V/cm, 13.0 mA. NP morphology was observed by transmission electron microscopy (Tecnai G^2^ 20 S-Twin, USA) at accelerating voltage of 80 kV.

### Fluorescence spectroscopy

We prepared CHCP**–**HSA mixtures at a molecule ratio of HSA to CHCP of 3.6∶1. CHP**–**HSA mixtures were similarly prepared. The mixtures were put in 2-mLeppendorf tubes, which were shaken with a rotating speed of 20 rpm at 37°C for 12 h. Fluorescence spectra and fluorescence intensities (FI) of free HSA and the NP-bound HSA were recorded by fluorescence spectrophotometry (Shimadzu RF-4500, Japan). The tryptophan chromophore in HSA molecule was excited at 280 nm and emission spectra were recorded at 290 to 450 nm [41]. Excitation slit width was 5 nm and emission slit width was 12 nm. Six NP solutions at different concentrations were mixed with HSA solution. The mixed solutions were transferred to 2-mLeppendorf tubes for 9-h interaction. The obtained samples were collected to measure fluorescence spectra in wavelength range of 290–450 nm. The fluorescence spectrum of pure HSA solution was used as a reference to determine binding constants according to Stern-Volmer analysis. Fluorescence quenching data was analyzed by the modified Stern-Volmer equation [42]:
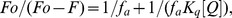
where *f_a_* is the fraction of accessible fluorophore (protein) to a polar quencher, *K_q_* is the Stern-Volmer quenching constant, *F o* and *F* are fluorescence intensities at 342 nm in absence and presence of quencher, and *[Q]* is the quencher concentration.

### CD analysis

CD spectra of free HSA and protein adsorbate after the addition of NPs were recorded at 200-to 250-nm wavelength by CD spectrometry (JASCO J-810, Japan) at 37°C with a 0.1 cm cuvette cell. The concentration of HSA was 1.0 mg/mL in all samples. CD spectra of NP**–**HSA complexes were also collected to determine α-helical content. The relative α-helical content within HSA was calculated as follows [43], [44]:
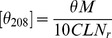


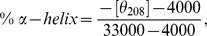
where [*θ_208_*] is the mean residue ellipticity in deg cm^2^ dmol^−1^ at 208 nm, *θ* is ellipticity measured at 208 nm, *M* is the molecular weight of HSA, *C* is the concentration of HSA (mg/mL), *L* is the length of cuvette cell (cm), and *N _r_* is the number of amino acids in the HSA molecule.

### *In vitro* drug release

Mitoxantrone-loaded NPs were prepared by a dialysis method [45] and their sizes were studied by dynamic light scattering. The encapsulation and loading capacity of NPs were measured and calculated as described [46]. Mitoxantrone release was studied *in vitro* by dialysis in phosphate buffered saline (PBS). Briefly, the solution of mitoxantrone-loaded NPs (2 mg/mL) was placed into Visking dialysis tubing (molecular weight cut off 12–14 kDa, USA) and dialyzed against the release media at 37°C in an air-bath shaker at 50 rpm. At predefined time intervals, the release media was collected and fresh release media was added. The released mitoxantrone was determined by UV spectrophotometry (UV-384 plus, Molecular Devices Corporation, USA) at 608 nm, and the cumulative release percentage (Q %) was calculated as described [38]. An amount of HSA solution (0.1 mg/mL) was added to the dialysis tube to measure drug release of 2 types of NPs. A weight ratio of Mitoxantrone to HSA of 1∶10 was prepared to obtain HSA**–**mitoxantrone attachment, and the mixture solution was dialyzed against 1000 mL distilled solution for 6 h to remove free mitoxantrone. The drug release of HSA**–**mitoxantrone attachment was determined as described above to further understand drug release of HSA-bound NPs.

## Results

### CHCP conjugate

**Figure 1:**
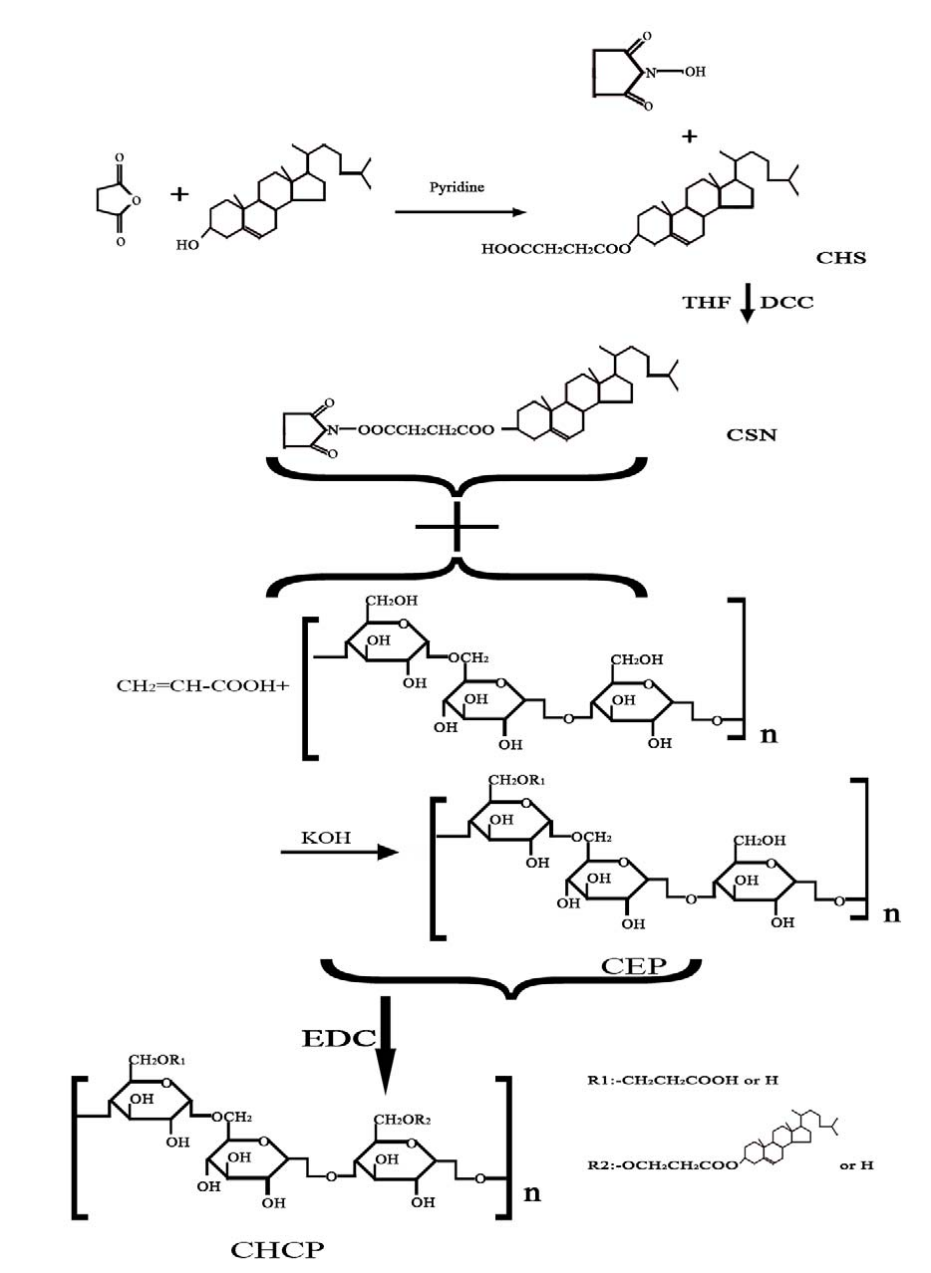
Schematic illustration for synthesis of CHCP conjugate.

**Figure 2:**
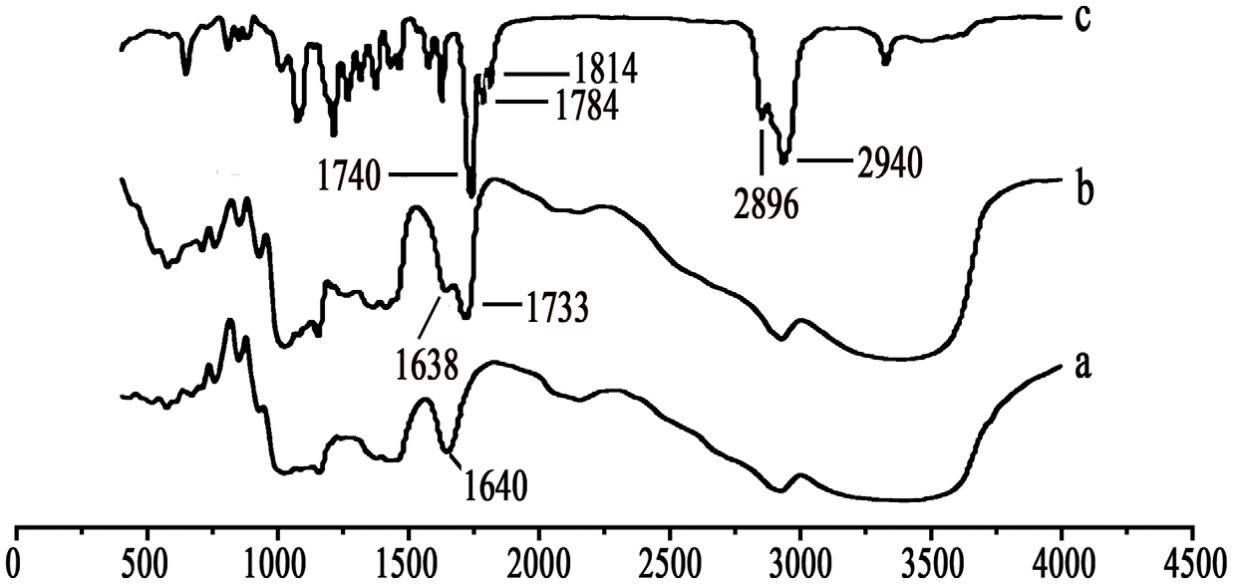
Infrared spectra of (a) pullulan, (b) CEP, and (c) CHS.

**Figure 3:**
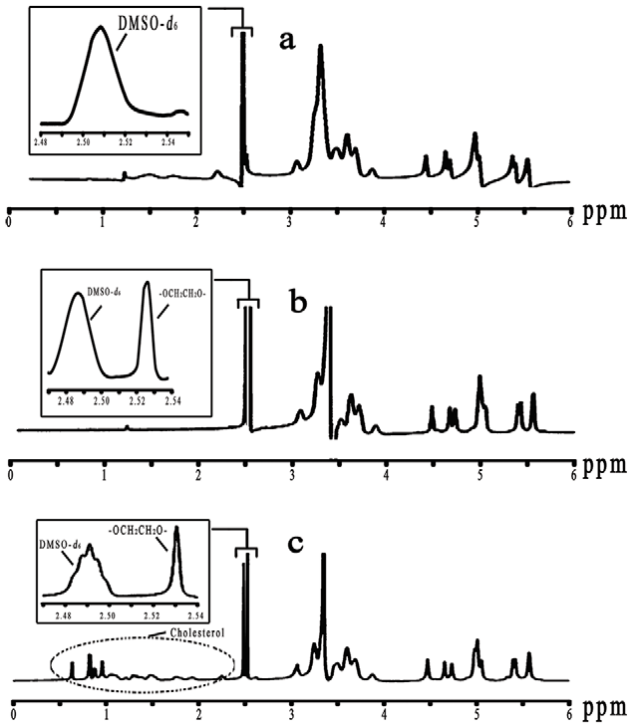
Nuclear magnetic resonance spectra of (a) pullulan, (b) CEP, and (c) CHCP.

Pullulan was carboxyethylated to yield CEP to improve solubility and bioactivity (Figure 1). Then cholesterol was covalently attached to CEP to produce a novel kind of polymeric amphiphile (Figure 1). Figure 2 shows FT-IR spectra of pullulan, CEP and CSN. As compared with pullulan, CEP show that besides an absorption peak at 1640 cm^−1^ (C-O stretch), the band at 1733 cm^−1^ (C = O) confirms that the carboxyethyl group was conjugated to pullulan. The peak assignment of CSN is as follows (in cm^−1^): 2940 (−CH_2_), 2896 (−CH_3_), 1814 and 1784 (C = O stretch of CO-N in NHS), and 1740 (C = O stretch of CO-O in CHS). According to the characteristic peaks described above, CSN was successfully synthesized by esterification reaction. Figure 3 shows ^1^H NMR spectra for pullulan, CEP and CHCP. As compared with pullulan, CEP showed signals at 2.4–2.6 ppm, belong to the carboxyethyl group. CHCP ^1^H-NMR spectra analysis allowed for identifying protons corresponding to pullulan chain at: 0.40–2.40 (the H signal of cholesterol), 2.49 (DMSO-*d_6_*), and 2.53 (2 methylene groups, -OCH_2_CH_2_O-) ppm. The characteristic proton signals of CHCP appear at 0–3.0 ppm. DS of carboxyethyl and cholesterol residues per 100 glucose units in pullulan could be calculated by the ratio of methylene protons (2.53 ppm) to sugar protons (C_1_ position of α-1, 6 and α-1, 4 glycosidic bonds, 4.68 and 5.00 ppm) with the following equation:
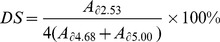
where A _∂2.53_ is the spectrum area under the characteristic methylene (hydrogen) peak, and A _∂4.68_ and A _∂5.00_ are spectrum areas under characteristic proton peaks of α-1, 6 and α-1, 4 glycosidic bonds, respectively. The DS values calculated from the spectra for carboxyethyl and cholesterol groups in the CHCP conjugate were 10.13% and 3.14%, respectively.

### CHP and CHCP NPs

**Figure 4:**
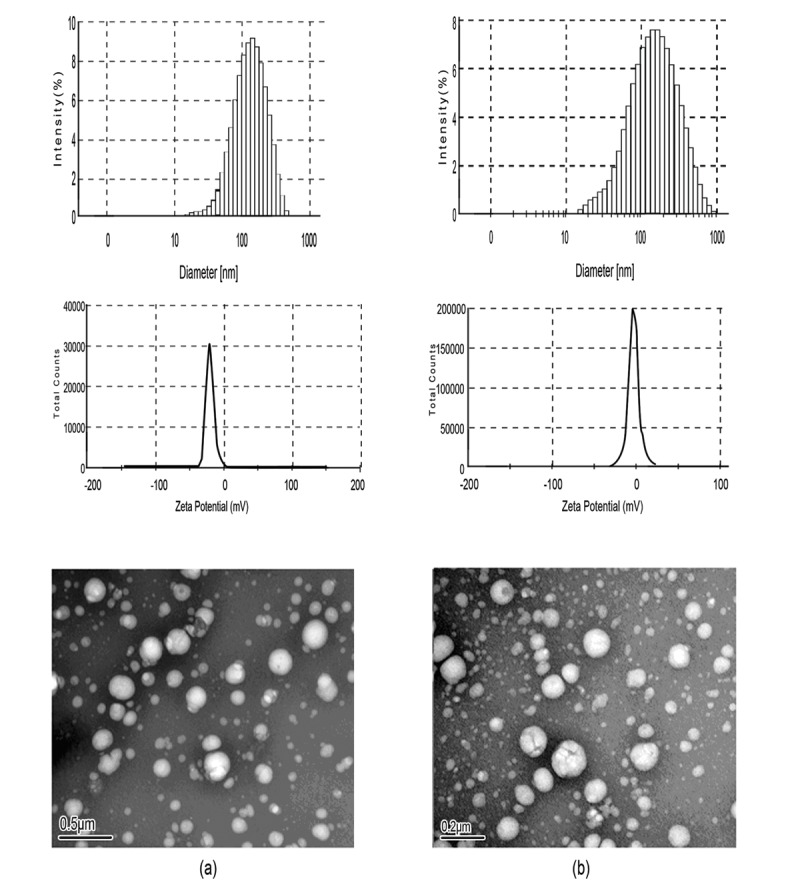
Size distribution, zeta potential, and transmission electron microscope images of (a) CHCP nanoparticles and (b) cholesterol-modified pullulan (CHP) nanoparticles.

CHP conjugates, composed of hydrophilic pullulan backbone and partly substituted hydrophobic cholesterol, can form a stable hydrogel NP in aqueous solution because of non-covalent association from intra-and/or intermolecular interaction among hydrophobic segments [47]. The morphologic features and sizes are controlled by the chemical composition of CHP, the molecular weight of pullulan and DS of cholesterol groups [35], [38], [48]. The increasing DS of cholesterol caused decreasing NP size because it enhanced the chance of hydrophobic interaction among hydrophobic pendant groups, which resulted in the formation of more compact hydrophobic cores [49], [50]. Pullulan (550 kDa) hydrophobically modified with cholesterol groups (DS = 3.4%) can form CHP aggregates with a mean diameter of 20∼30 nm [35]. We chose pullulan at 200 kDa to synthesize CHP conjugates with 3.11% DS of cholesterol groups. The mean size, size distribution, zeta potential, polydispersity index and morphologic features of NPs are shown in Figure 4 and Table 1. The mean size of formed CHP NPs was 110.8±4.6 nm, with polydispersity index 0.267±0.018.

**Table 1:** Characterization of nanoparticles in distilled water.

Sample	D^a^ (nm)	PI^a^	ZP^b^ (mV)
CHP	110.8±4.6	0.267±0.018	−1.21±0.12
CHCP	148.6±3.2	0.189±0.023	−19.9±0.23

a Average diameter (mean value ± S.D) determined by dynamic laser light-scattering with three times. (PI: polydispersity index).

b Zeta potential (mean value ± S.D) determined by zeta potentiometer with three times.

Besides affected molecular weight and DS of cholesterol, the sizes were affected by carboxyethyl groups of polymeric amphiphiles. The mean size of CHCP self-aggregated NPs prepared by probe sonication was 148.6±3.2 nm with polydispersity index 0.189±0.023. The DS for cholesterol moiety and molecular weight were similar between CHP and CHCP, so the only difference in chemical structure was in carboxyethyl group values. The hydrophobically self-aggregated force of CHCP molecules with the interference of ionic (COO^−1^) repulsion force led to the formation of larger-sized NPs with a loose structure in aqueous solution. CHCP NPs, with zeta potential −19.9±0.23 mV as compared with −1.21±0.12 mV for CHP NPs, suggested that the negatively charged carboxyethyl groups in CHCP molecules contributed to particle surface charges. Transmission electron microscopy revealed that these NPs were regularly spherical in shape.

### Fluorescence analysis of NP–HSA formation

Serum proteins such as albumin, apolipoprotein, fibrinogen and immunoglobulin can bind intravenously injected NPs that enter the body [2], [19]. We chose HSA to study the NP**–**protein interaction because it is be one of the prominent plasma proteins with high affinity to many exogenous and endogenous substances. NPs typically bind HSA and show different binding characteristics depending on hydrophobicity and radius of curvature [16]. NP surface properties, such as surface hydrophobicity and charges, also play a significant role in determining HSA binding [25].

**Figure 5:**
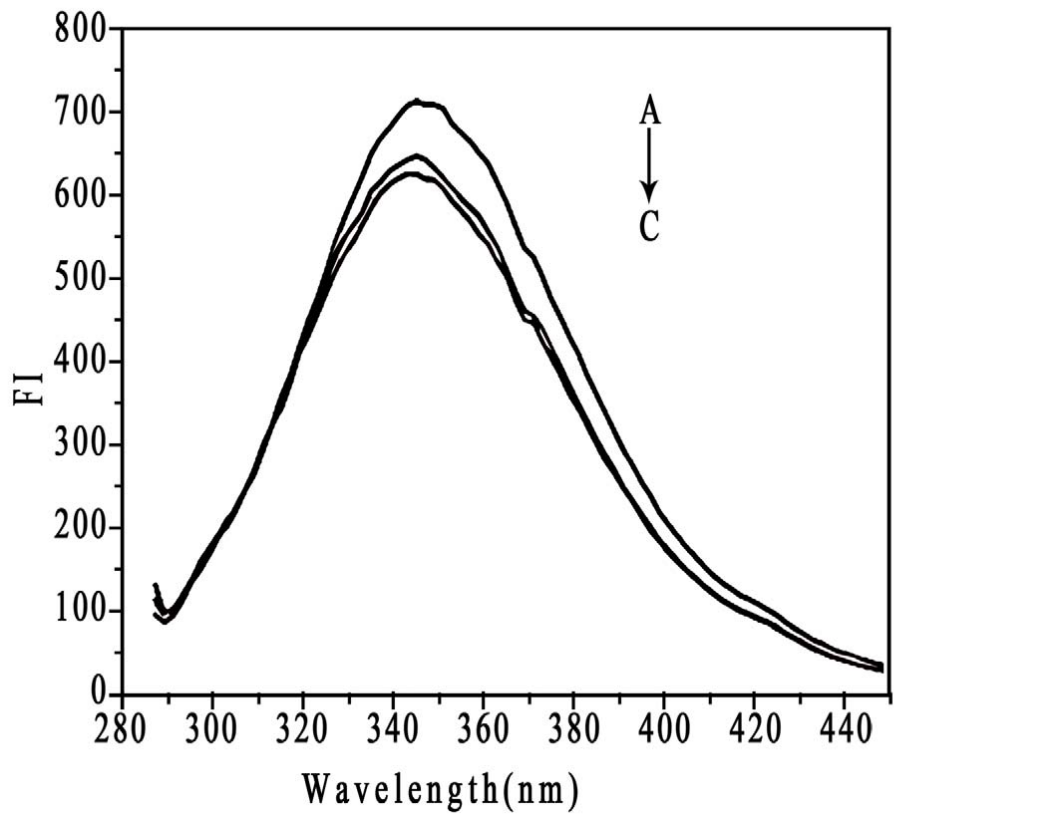
The fluorescence spectra of human serum albumin (HSA, 1.5×10^−5^ mol/L) (A) in the absence and (B) presence of the mixture of CHCP, and (C) CHP with the same concentration (4.2×10^−6^ mol/L).

**Figure 6:**
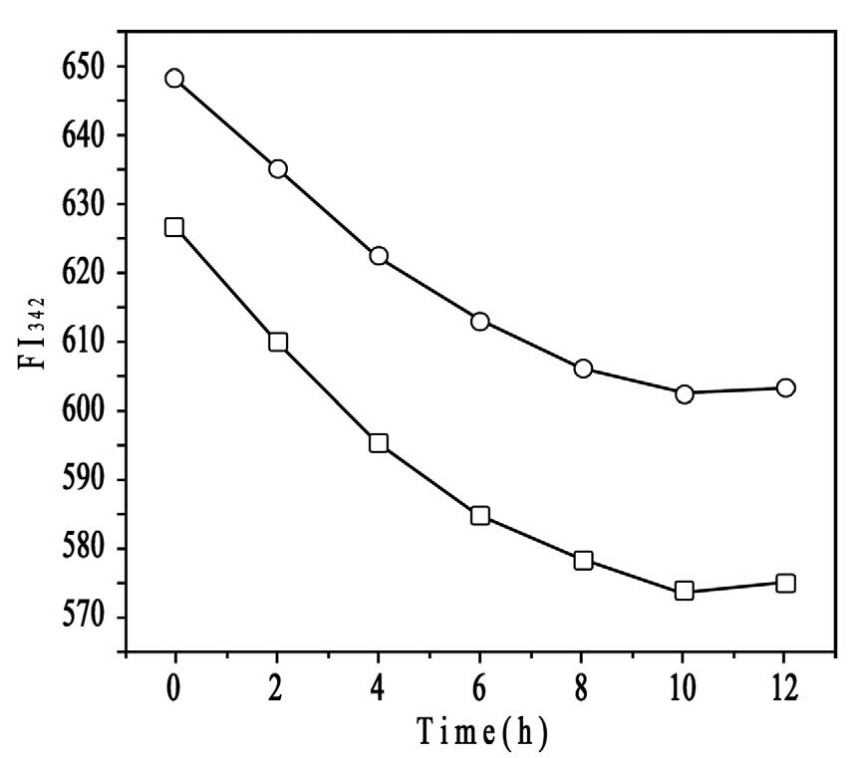
Emission intensity of HSA upon interaction with CHCP nanoparticles (—○—) and CHP nanoparticles (—□—) at 342 nm as a function of time.

We studied the interaction between HSA and 2 types of NPs with different surface charges by fluorescence spectroscopy. HSA contains a single polypeptide of 585 amino acids with only one tryptophan (Trp 214), which dominates the fluorescence spectra in the UV region [41]. When other molecules interact with HSA, tryptophan fluorescence may change, depending on the impact of such interaction [51]. NP**–**protein interaction is often monitored by fluorescence quenching because of the sensitivity and ease of use of the technique [52], [53]. Free HSA and NP**–**HSA mixture solutions give fluorescence emission intensities with a maximum value at 342 nm due to the tryptophan residue in HSA molecule (Figure 5). HSA fluorescence intensity was quenched by 2 kinds of NPs. CHP NPs more strongly quenched HSA fluorescence intensity than did CHCP NPs, which indicated that besides particle hydrophobicity, surface charge governs fluorescence quenching with the NP**–**HSA interaction. Fluorescence emission intensities of CHP**–**HSA and CHCP**–**HSA at 342 nm as a function of time revealed that fluorescence intensity decreased with the interaction and reached a plateau at 9–12 h reaction (Figure 6). Because the complexation between BSA and CHP NPs involved a two-step process [35]–[37], the NP**–**HSA complex probably also underwent this process, as reflected in fluorescence intensity gradually decreasing. The initially rapid decrease in fluorescence intensity was due to the fast adsorption of HSA to NPs, whereas the slowly decreasing is due to the gradual complexation between them. Finally, the NP**–**HSA complex was completely formed by 9–12 h, with no further change in fluorescence intensity. The fluorescence intensity decreasing for CHCP**–**HSA complex was similar to that for CHP**–**HSA, except that the degree of decrease was weaker for CHCP than CHP. The complex formation was driven by a hydrophobic force between cholesterol groups in CHP and aromatic amino acids in HSA. NPs with a hydrophobic cholesterol core and hydrophilic polysaccharide shell showed a spherical structure, which determined the NP**–**HSA complexation was complicated and slow. Hydrophobic interaction was the drawing force for HSA adsorption to NPs. However, polysaccharide chains of the outer shell were interferential for the further and completed complexation. As for CHCP NPs, HSA showed additional interference in complex formation because of charge repelling force from domains of negative charge and surface charge (COO^−1^) of NPs. This finding may explain the gradual decrease in fluorescence intensity (Figure 6).

**Figure 7:**
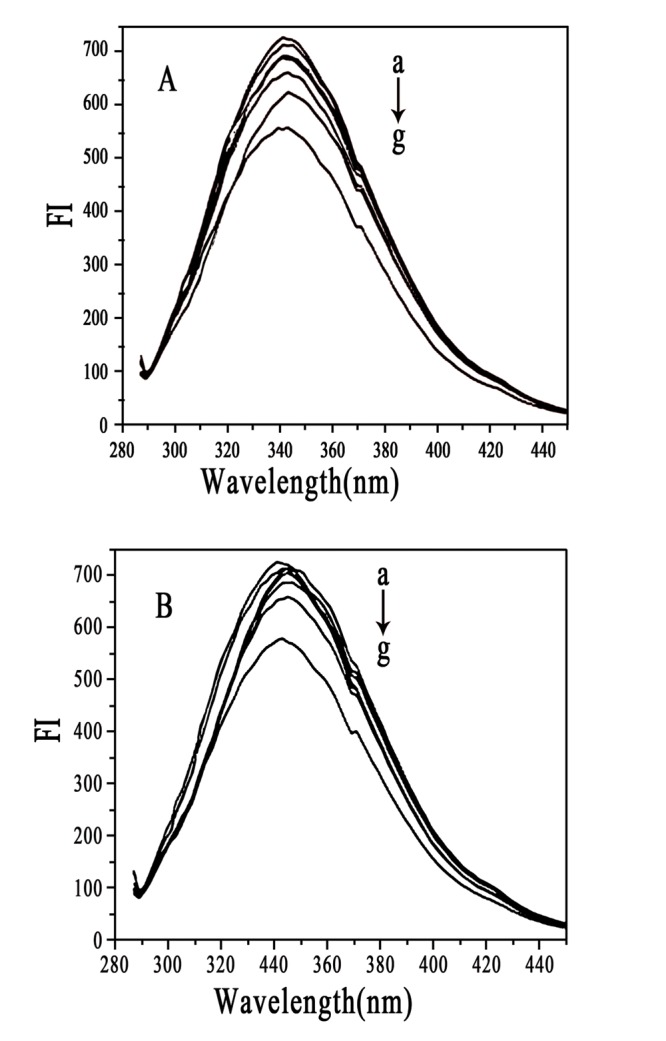
Fluorescence spectra of HSA (1.5×10^−5^ mol/L) in the presence of (A) CHP and (B) CHCP with different concentrations: (a) 0, (b) 2.07×10^−7^, (c) 3.31×10^−7^, (d) 4.14×10^−7^ , (e) 8.28×10^−7^, (f)20.7×10^−7^, and (g) 41.4×10^−7^ mol/L.

**Figure 8:**
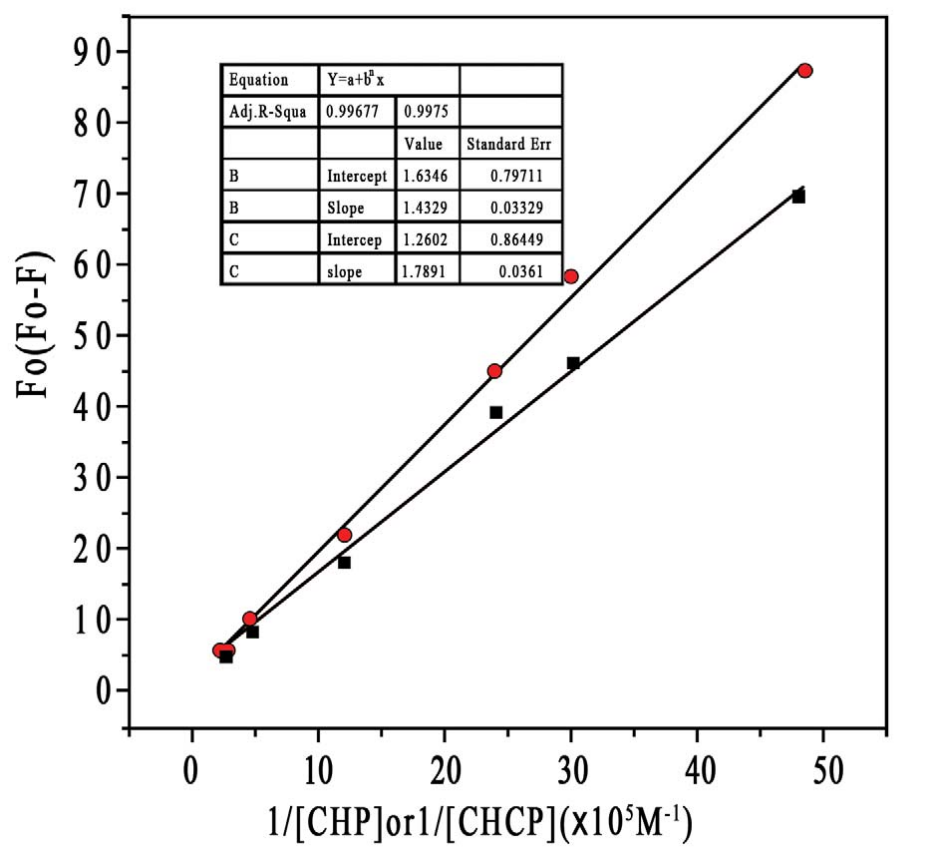
Plots (n = 6) of Fo/(Fo −F) versus 1/[CHP] (—▪—) and 1/[CHCP] (—•—). The concentration of HSA was 1.5×10^−5^ mol/L.

A previous study suggested that NP**–**protein interaction depended on NP surface composition and size but also protein identity [25]. Rates of both association and dissociation are higher for HSA than apolipoprotein A-I and many other plasma proteins [19]. HSA may predominate on the particle surface at short times and is subsequently displaced by lower-abundance proteins with higher affinity [15], [17]. The molecular composition of NPs also strongly influences the exchange rates. The HSA affinity is lower for the more hydrophobic than more hydrophilic particles [16]. To explore the effect of NP surface charge on HSA binding force, we used fluorescence quenching to obtain the binding parameters by Stern-Volmer analysis. HSA fluorescence intensity decreased with increasing CHP concentration from 2.07×10^−7^ to 4.14×10^−6^ M (Figure 7), so CHP NPs quenched the fluorescence of tryptophan residues, as did CHCP NPs. The plots of *F o/(F o −F)* versus *1/[CHP]* or *[CHCP]*, slope and intercept are shown in Figure 8. Quenching constants were calculated from the fluorescence data and found to be *K* (1.14×10^5^) and (0.70×10^5^) for CHP and CHCP, respectively. The *f _a_* values (0.612 and 0.794) corresponded to CHP and CHCP, indicating that part of the tryptophan residues were involved in the NP**–**HSA complex. From *K* values, we inferred that NP surface charges greatly influenced HSA fluorescence quenching. Assuming that the observed changes in fluorescence result completely from the interaction between NPs and HSA, the quenching constant (*K_q_*) can be taken as a binding constant (*K_b_*) of the complex formation [51]. Therefore, the binding force may be stronger with the formation of CHP**–**HSA than CHCP**–**HSA. Thus, the affinity of HSA was lower for NPs with negative charges, and there will be competitive binding as these 2 NP types are injected into the body. CHP NPs may have a higher degree of surface coverage with HSA because of its higher affinity. The amount and presentation of protein on the NP surface causes numerous biological responses and influences particle biodistribution, clearance, and cell uptake [19], [54]–[55]. Therefore, NPs engineered with certain surface compositions will be vital for their *in vivo* efficacy due to the effect on protein adsorption.

### Secondary structure of bound HSA

**Figure 9:**
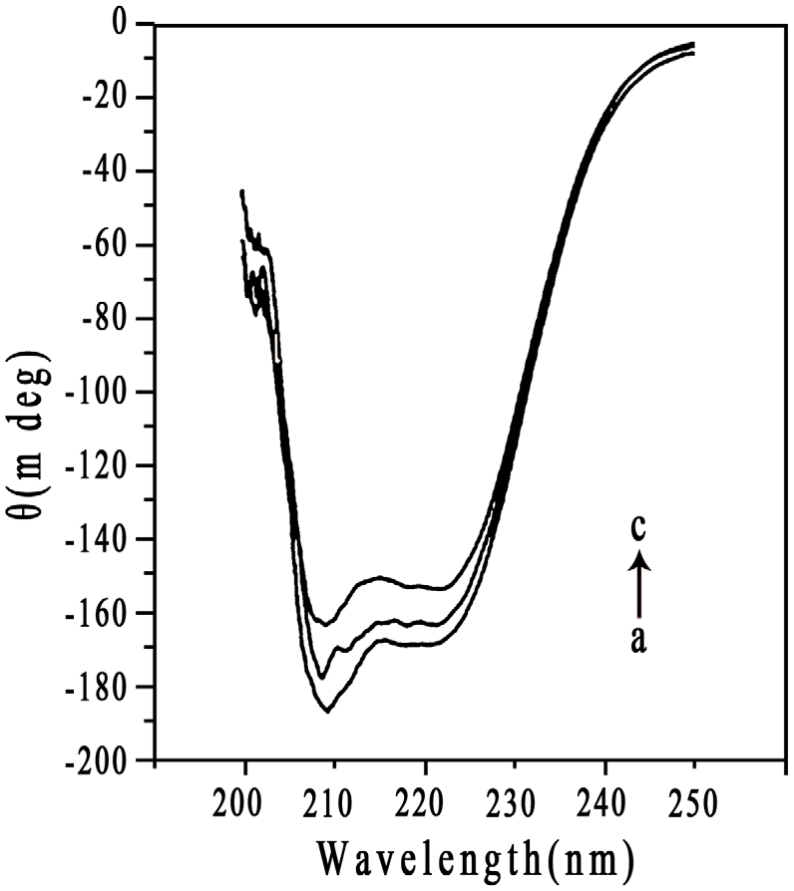
CD spectra of HSA in the (a) without (b and c) with CHP and CHCP nanoparticles in solution at 37°C.

NPs coated with proteins in biological fluids may have nanotoxicologic effects because of NP- induced alteration in protein conformation and function [56]–[58]. To identify alteration in HSA secondary structure after the addition of NPs, CD spectroscopy was employed due to its sensitivity to the change in protein structure. The CD spectra of free HSA and NP**–**HSA adsorbate in the near UV spectra are in Figure 9. The spectra for HSA showed a negative peak at 208 nm, characteristic of the α-helical structure [59]. The α-helical content of free HSA was 57.2%. After adsorption to NPs, the α-helical content of HSA decreased to 49.2% for CHP and 53.6% for CHCP. Of note, the gradual complexation between NPs and HSA altered the protein structure. We recorded ellipticities of NP**–**HSA complexes to obtain the α-helical content of the complexed HSA. After allowing complex formation for 12 h, the α-helical content of HSA decreased to 43.6% for CHP and 49.3% for CHCP. The alteration in fluorescence intensity was related to the impact on protein conformation upon the interaction between HSA and exotic molecules [51]. The maximum fluorescence intensity gradually decreased during the NP**–**HSA complexation, indicating that the α-helical content decreased. Besides depending on interaction time, albumin conformation was altered to a great degree by the addition of NPs with higher hydrophobicity [35]. In this work, the binding force and the complexation process were immensely influenced by NP surface charge. From preliminary adsorption to the final complex, the α-helical content of HSA decreased less on the interaction with CHCP than CHP NPs. Thus, the alteration in albumin conformation by NPs depends on particle size, hydrophobicity, surface charge, and interaction time, which have implications for controlling the change in protein conformation and function in the design of suitable nanocarriers for drug delivery.

### Drug release analysis

**Figure 10:**
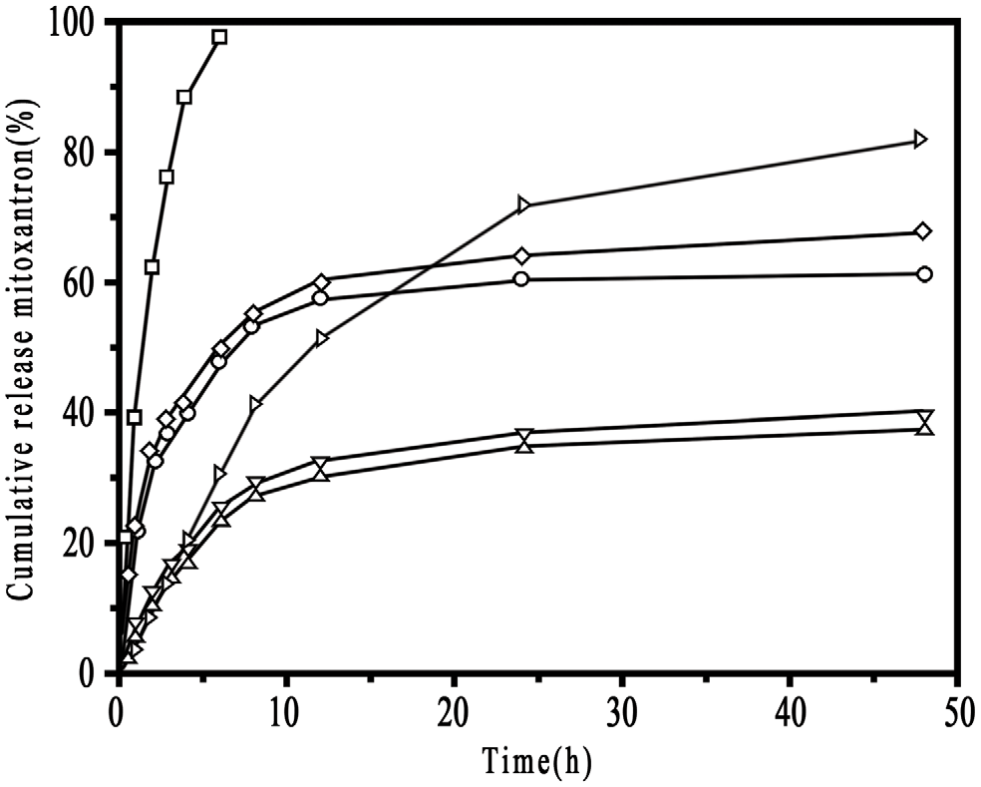
The release of mitoxantrone in phosphate buffered saline at 37°C in vitro (□, free mitoxantrone; ○, CHP; ◊, CHCP; ▹, HSA-mitoxantrone; ▽, CHCP-HSA; Δ, CHP-HSA).

We measured encapsulation (%) and loading capacity (%) to compare drug-loaded characteristics between 2 types of NPs (Table 2). Encapsulation and loaded capacity were about 58.6% and 7.12% for CHP NPs versus 50.8% and 6.14% for CHCP NPs. The capacity for mitoxantrone loading was greater for CHP than CHCP NPs. The mean diameters of CHP and CHCP NPs were approximately 168.6 nm and 192.7 nm, respectively. After drug entrapment, these NPs showed greater diameter than did free NPs. Mitoxantrone release behavior of NPs and HSA-complexed NPs were studied *in vitro* in PBS. Drug release was shown in Figure 10. Free mitoxantrone has a fast release into outer media, with greater than 97.2% of the drug being released after 6 h. Drug release from NPs involves 2 processes: rapid and slow release, which can be attributed to hydrophobic drugs trapped by NPs with a surface adsorption and core loading [8], [38]. For CHP NPs, we observed rapid release, mainly from the particle surface, up to 8 h, when 52.6% of the drug was released. After 8 h, mitoxantrone was released continuously up to 48 h, mainly from the particle core, reaching a percentage of cumulative release of about 62.8%. Similar release characteristics were observed for CHCP NPs: a rapid release of 53.3% and a total release of 68.2%. The drug amount of HSA**–**mitoxantrone attachment was (38.6±1.73) % to the total amount of initial mitoxantrone. The release profile of HSA**–**mitoxantrone attachment exhibited a very steady sustained-release pattern, which showed drug release of 41.8% after 8 h and 80.6% after 48 h. Drug release was slower as drug-loaded NPs bound to HSA. The total drug release of NPs after 48 h was 37.4% and 38.6% for the bound CHP and CHCP, respectively. The slower release of the complexed NPs could have 2 explanations. First, HSA molecules could quickly bind to NP surface and but be slowly complexed into the hydrogel matrix of NPs, which formed a surface steric for drug release from mitoxantrone-loaded NPs. Second, mitoxantrone could bind HSA through hydrophobic interaction, electrostatic interaction and hydrogen bonding with an association constant of the order of 10^5^ [22], [60]. Released mitoxantrone from NPs would bind to the bound HSA. Diffusion of mitoxantrone from within a dialysis tube into PBS was sustained by these 2 kinds of binding. The total drug released was greater with CHCP than CHP NPs; however, this difference was minor as compared with drug released with the bound CHP and CHCP. The main reason is that the HSA complexation caused a slower release by the steric hindrance and repeated binding.

**Table 2:** Characterization of mitoxantrone-loaded nanoparticles.

Sample	Drug/carrier (w/w)^a^	Diameter (nm)^b^	Encapsulation (%)^c^	Loading capacity (%)^d^
CHP	1/10	168.6±3.3	58.6±3.14	7.12±0.24
CHCP	1/10	192.7±4.2	50.8±2.26	6.14±0.16

a Mitoxantrone/CHP or CHCP NPs (mg/mg).

b Size and size distribution (mean value ± S.D) determined by dynamic light scattering with three times.

c Encapsulation efficiency (mean value ± S.D) determined by UV spectrophotometry at 608 nm with three times.

d Loading capacity of NPs (mean value ± S.D) determined by UV spectrophotometry at 608 nm with three times.

## Discussion

We characterized the process of NP**–**HSA interaction, the effect of particle surface charge on HSA complexing, the alteration in HSA conformation by NP complexation, and the drug release of HSA-bound NPs. Formation of the NP**–**HSA complex was driven by a hydrophobic force between cholesterol groups of the particle core and the aromatic amino acids of the hydrophobic domain. After mixing, HSA was rapidly adsorbed to NP surface by hydrophobic interaction with surface cholesterol units. The adsorbed HSA continued to be drawn off due to the hydrophobic forces from cholesterol units in the particle core. The adsorbed HSA gradually moved into the core, while overcoming the steric hindrance of polysaccharide chain in the NP shell. As a result of the balance between the hydrophobically drawing force and the hydrophilic polysaccharide-chain resisting force, HSA molecules entered the particle core to become hydrophobically bound to cholesterol groups, thus completing the NP-HSA complex.

For CHCP, the HSA complexation was a slow and complicated process, involving particle surface adsorption and gradual insetting into the core. During this process, the CHCP**–**HSA complex formation suffered from interference of the negative repulsive force between NPs and HSA. It involved a weaker binding force to form the looser NP**–**HSA complex. In NP**–**HSA complex process, the α-helical content decreased, suggesting the peptide chain unfolding. The unfolding of the peptide chain began with particle adsorption and developed during the complex process. The gradual increase in unfolded peptide chain can be ascribed to the drawing force of hydrophobic attraction to the particle inner and the resisting force of the outer hydrophilic polysaccharide chain.

**Figure 11:**
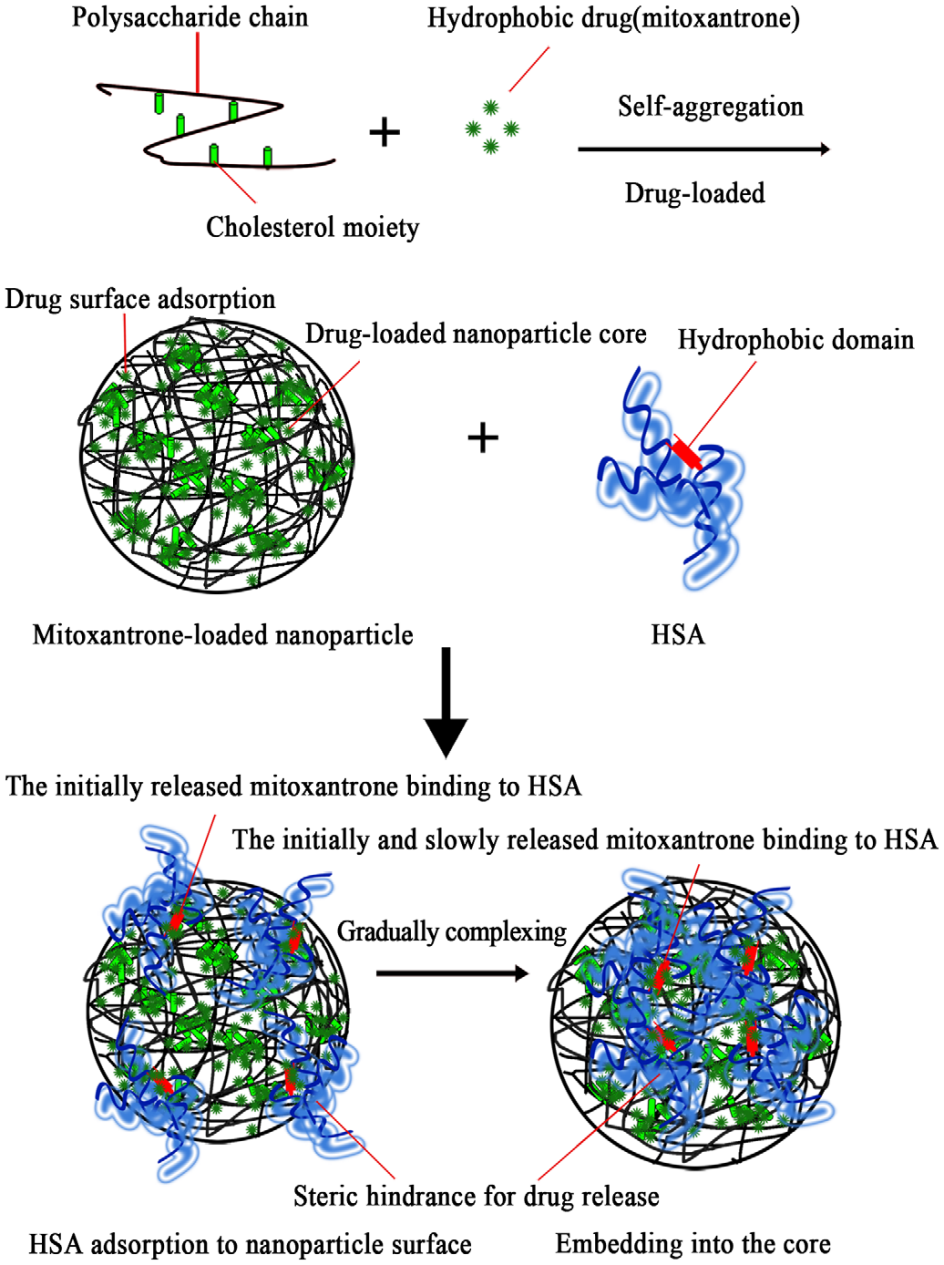
Effect of HSA complexation on drug released from nanoparticles.

Figure 11 shows the self-aggregated process of drug-loaded NPs and the effect of HSA complexation on drugs released from NPs. CHP self-aggregated to form stable hydrogel NPs with a hydrophobic core and hydrophilic shell, in which pullulan chains are noncovalently crosslinked by associating cholesteryl moieties [9], [47]. Cholesterol modified polysaccharides can incorporate the small-molecule hydrophobic drug mitoxantrone during the self-assembled process of NPs [38]. The albumin complexation influences the release behavior of loaded proteins from CHP NPs [33], [34]. In this study, we show the release characteristics of mitoxantrone from CHP NPs in NP**–**HSA complexation. Initially, the drug released from the NP surface was adsorbed to HSA. Then, the drug**–**HSA complex rapidly bound to the particle surface. Subsequently, the drug released from the NP core was still adsorbed by the complexed HSA. Therefore, the HSA-bound NPs showed dual sustained drug release. In addition, mitoxantrone-loaded NPs coated with the adsorbed or completely complexed HSA molecules inhibited the drug release by a steric hindrance effect.

If albumin can not bind NPs, the amount of drug released from NPs would be enhanced because the fast-released drug is adsorbed to HSA. The subsequent decrease in mitoxantrone concentration in the vicinity of NPs favored further release. Of note, HSA binding or not to NPs determines the enhancing or inhibiting effect on initially sustained drug release, which depends on whether the chosen model drug can be adsorbed to HSA. Binding of a drug to HSA is important in determining its metabolism, distribution, and elimination from the circulation. HSA binding influences organ distribution and clearance of NPs, and thus changes the *in vivo* efficacy and therapeutic efficiency of the loaded drugs. In this paper, we reveal NPs trapping mitoxantrone, the drug release of mitoxantrone-HSA, and HSA complex formation with NPs. HSA could bind NPs as well as the released drug, which promoted the initially sustained drug release. Ultimately, properties such as size, hydrophobicity and surface charge of NPs are vital for realizing this promising area of nanotechnology in drug delivery because of their influence on protein binding and drug release.

In summary, we prepared novel self-aggregated NPs from polymeric amphiphiles, CHCP conjugates. The negatively charged carboxyethyl groups and hydrophobic cholesterol groups played a predominant role in the formation of NPs with a certain size and morphologic features. NP hydrophobicity and surface charge determined the HSA binding force and greatly influenced albumin conformation during the complex formation. Compared to CHP NPs, CHCP NPs had a lower affinity to HSA and induced α-helical content decreasing with a smaller degree from the initial adsorption to the final complex. The HSA binding promoted the initially sustained drug release from the loaded NPs. Further study of NP interaction with biological bodies is vital to fully understand the potential application of these drug carriers in medicine.
